# Solitary synchronous gastric metastasis of renal cell carcinoma

**DOI:** 10.1002/iju5.12239

**Published:** 2020-11-06

**Authors:** Shigeki Koterazawa, Jun Watanabe, Yuichi Uemura, Masayuki Uegaki, Toshiaki Shirahase, Yoji Taki, Yasushi Adachi, Michimsasa Ueda, Shouichi Fukui

**Affiliations:** ^1^ Department of Urology Toyooka Hospital Toyooka Hyogo Japan; ^2^ Department of Pathology Toyooka Hospital Toyooka Hyogo Japan; ^3^ Department of Gastroenterology Toyooka Hospital Toyooka Hyogo Japan; ^4^ Department of Urology Public Interest Incorporated Foundation Tango Central Hospital Kyotango Kyoto Japan

**Keywords:** endoscopic resection, nephrectomy, metastasectomy, renal cell carcinoma, solitary synchronous gastric metastasis

## Abstract

**Introduction:**

There have been some reports describing metastasis to the stomach from renal cell carcinomas. However, there are few reports describing solitary synchronous gastric metastasis of renal cell carcinomas.

**Case presentation:**

The patient was a 70‐year‐old woman who underwent an upper gastrointestinal endoscopy to examine her progressive weight loss. There was a submucosal tumor in the stomach, which was biopsied. The gastric tumor was pathologically proven to be a metastatic clear cell renal cell carcinoma. Furthermore, contrast‐enhanced computed tomography showed right renal cell carcinoma invading the renal vein (cT3aN0M0). The patient underwent right radical nephrectomy and endoscopic resection for the treatment of the primary renal cancer and the gastric metastatic lesion, respectively. The resected specimen of the stomach had a clear resection margin.

**Conclusion:**

Endoscopic resection for early stage gastric metastatic lesions of renal cell carcinomas is a reasonable approach because it is a minimally invasive surgical technique.

**Abbreviations & Acronyms**
CT: computed tomography
RCC: renal cell carcinoma

**Keynote message**

We experienced a rare case of solitary synchronous metastasis to the gastric mucosa of an RCC patient. For patients with resectable metastatic lesions of RCCs, complete metastasectomy should benefit patients’ survival. Moreover, endoscopic resection for an early stage gastric metastatic lesion is a reasonable approach because it is minimally invasive surgical technique.

## Introduction

RCCs metastasize at the time of diagnosis or during the course of treatment in approximately half of all cases. The usual sites of RCC metastasis include the lungs, bones, liver, and brain.^1^ There are few reports of metastatic RCCs to the stomach, which make cases of solitary synchronous gastric metastases of RCCs extremely rare. Here, we present a patient with a solitary synchronous gastric metastasis of RCC diagnosed with upper gastrointestinal endoscopy and CT. The patient underwent right radical nephrectomy and endoscopic resection for the treatment of the primary renal cancer and the gastric metastatic lesion.

## Case presentation

**Fig. 1:**
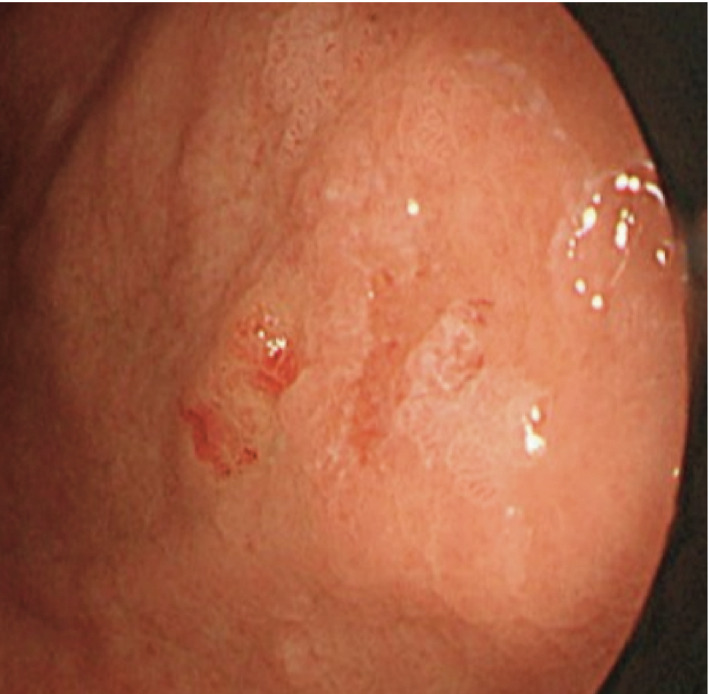
Upper endoscopy findings.

**Fig. 2:**
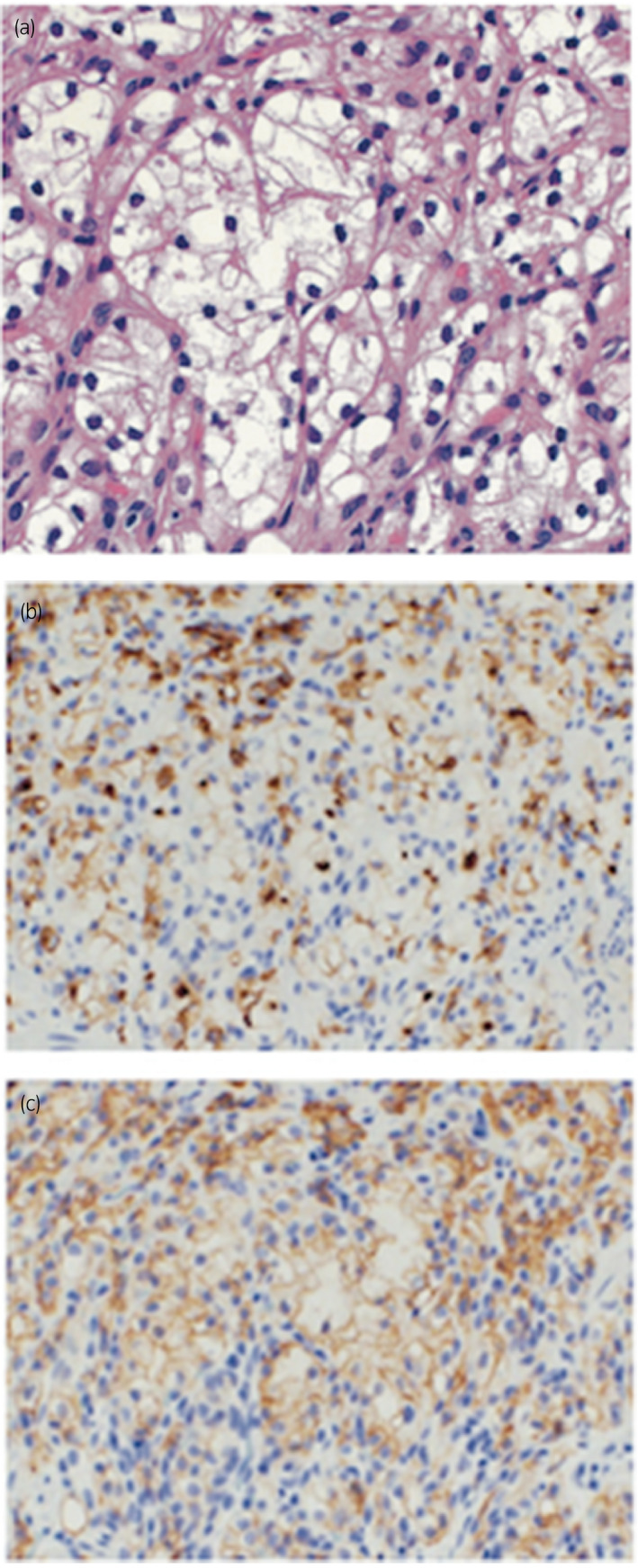
The biopsy specimen: (a) hematoxylin and eosin, (b) CD10, and (C) CAIX.

The patient was a 70‐year‐old woman who had experienced loss of appetite for several weeks. Upon physical examination, the abdominal tenderness and masses were absent. Laboratory data revealed no abnormalities other than an increase in C‐reactive protein (3.41 mg/mL). Upper gastrointestinal endoscopy showed a single 5‐mm reddish lesion in the posterior wall of the mid‐body of the stomach (Fig. 1). A biopsy of this lesion showed tumor cells with clear cytoplasm with prominent nucleoli. Immunohistochemical staining was positive for CD10 and CAIX. RCC antigen stain was focally positive. Histopathology and immunohistochemical staining were consistent with metastatic RCC of the clear cell type (Fig. 2). Endoscopic ultrasonography revealed the depth of the lesion was limited to the mucosal and submucosal layers.

**Fig. 3:**
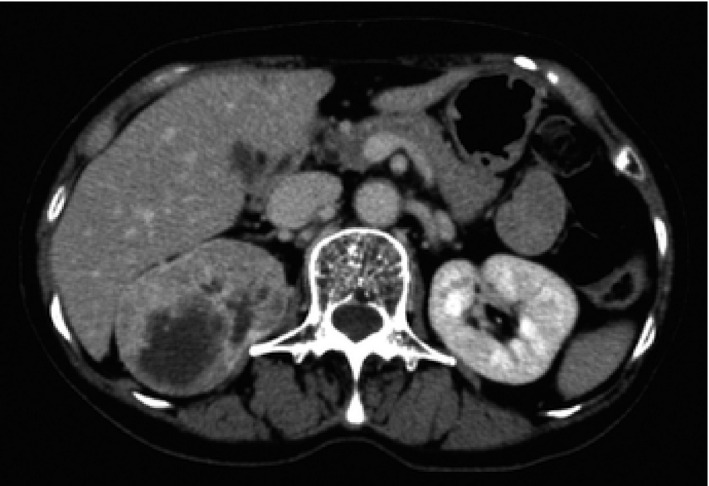
CT shows a large mass with early enhancement in the right kidney with tumor thrombus in the right renal vein.

**Fig. 4:**
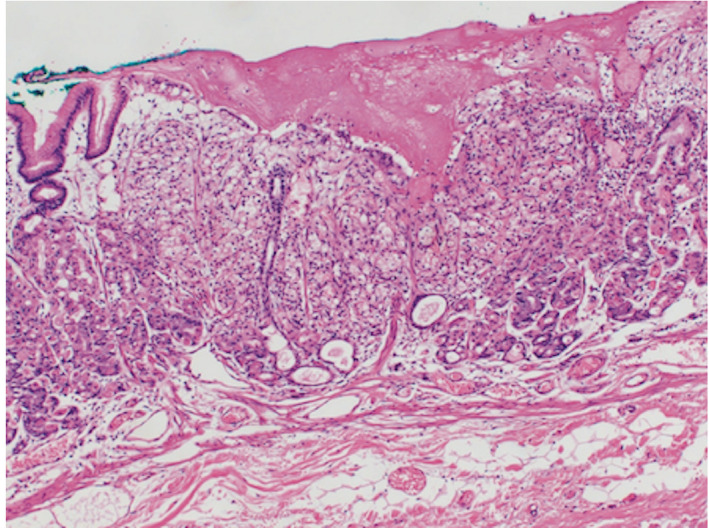
The resected specimen was approximately 3 mm in diameter with a clear resection margin.

Enhanced CT showed a mass, approximately 6.5 cm in diameter with early enhancement in the right kidney with a tumor thrombus in the right renal vein, but there was no evidence of lymph node enlargement or a metastatic lesion (Fig. 3). The patient was diagnosed with right RCC (cT3aN0M1) and underwent right radical nephrectomy. The tumor existed in the upper pole of the kidney and showed a well‐defined mass that was 42 × 40 × 40 mm in size. The tumor was diagnosed as clear cell type and had invaded the perirenal fat. Therefore the RCC was staged as pT3apN0M1. One month after the nephrectomy, the patient underwent endoscopic submucosal resection for the treatment of the gastric metastatic lesion. The resected specimen was approximately 3 mm in diameter with a clear resection margin, which was consistent with metastatic clear cell RCC (Fig. 4). There was no recurrence at 4 months after the endoscopic resection.

## Discussion

Metastatic gastric cancer is a rare disease; in fact, analysis during autopsy shows that metastatic gastric cancer occurs in 5.4% of all cancer patients. Particularly, melanoma, lung cancer, and breast cancer are common in primary cancers.^2^ With regard to metastatic RCC, gastric metastasis is rare (0.65%). The most frequent sites of metastasis include the lung, bone, liver, and brain.^1^, ^3^ Upper gastrointestinal endoscopy findings of metastatic gastric cancer include polyp‐like lesions, ulcerative lesions, and minor erosion. Additionally, metastatic gastric cancer is characterized by metastasis to the submucosa.^2^

Arakawa *et al*. reported 54 cases of gastric metastasis of RCC.^4^ In general, patients were more likely to be male (78%). The average time to detection of gastric metastasis from detection of the primary RCC lesion is 6.7 years (range 0–23 years). At the time of gastric metastasis diagnosis, 39 patients had multiple metastases in other organs and 15 patients were diagnosed with solitary gastric metastasis. Solitary metastatic gastric cancer was detected only in three patients at the same time of diagnosis of the RCC. Metastasectomy as a local treatment for gastric metastases includes laparotomy or endoscopic resection. There were 31 out of 54 patients who underwent metastasectomy through laparotomy or endoscopic resection for gastric metastases. Laparotomy and endoscopic resection were selected in 21 and 10 patients, respectively. In the 21 patients who underwent laparotomy, the average size of the gastric lesions was 3.0 cm (1–7 cm), whereas in the 10 patients who underwent endoscopic resection, it was 1.9 cm (0.6–5 cm). Of the 15 patients with solitary gastric metastasis from RCC, seven were treated with laparotomy, five with endoscopic resection, two with systemic therapy including a molecularly targeted drug or chemotherapy, and one with a combination of endoscopic resection and a molecularly targeted drug. Of the three patients with solitary synchronous gastric metastasis, two were treated with laparotomy and one was treated with endoscopic resection.

Treatment choices for metastatic RCC have recently been increasing, along with the introduction of molecularly targeted drugs and immune checkpoint inhibitors. In the treatment for local metastases of RCC, no general therapeutic strategy has been established. However, Dabestani *et al*. concluded that complete resection of metastases benefits patients’ overall survival and cancer‐specific survival.^5^ It has also been reported that patients at a poor risk for Memorial Sloan Kettering Cancer Center classification and International Metastatic RCC Database Consortium could benefit less from metastasectomy,^6^ therefore, the decision of local treatment for metastases of RCC should be made on the bias of systemic status.

It is debated whether to perform local treatment for metastatic gastric cancer. Progression of metastatic cancer in the stomach can lead to gastric bleeding, abdominal pain, and dysphagia, which may shorten survival, therefore, local treatment for metastatic gastric cancer may be important.^7^ These symptoms including bleeding, abdominal pain, and dysphagia have also been reported in gastric metastasis of RCC.^8^ The treatment for metastatic gastric cancer should be selected so that the surgical margins of the metastases are preserved. Endoscopic resection, which is minimally invasive, is effective for submucosal tumors in the early stages, such as in this patient, therefore, selection of appropriate cases is important.

In summary, we experienced a rare case of solitary synchronous gastric metastasis from an RCC, which was treated with nephrectomy and endoscopic resection. Although it is discussed, for patients with resectable metastases of RCC, it is important to determine whether complete resection of metastatic lesions benefits a patient’s life expectancy. Moreover, endoscopic resection for gastric metastasis is a reasonable option for minimal invasiveness.

## Conflict of interest

The authors declare no conflict of interest.
